# The moderating effects of parental psychological control on the relationship between unsociability and socio-emotional functioning among Chinese children

**DOI:** 10.3389/fpsyg.2024.1308868

**Published:** 2024-03-05

**Authors:** Hong Zheng, Yihao Hu, Yuchen Cao, Ran Li, Nan Wang, Xi Chen, Ting Chen, Junsheng Liu

**Affiliations:** ^1^Shanghai Changning Mental Health Center, Shanghai, China; ^2^School of Psychology and Cognitive Science, East China Normal University, Shanghai, China; ^3^Zhuhai No. 2 High School, Zhuhai, China; ^4^Xinhua Hospital, School of Medicine, Shanghai Jiao Tong University, Shanghai, China

**Keywords:** unsociability, parental psychological control, socio-emotional functioning, Chinese children, moderating effect

## Abstract

**Introduction:**

There have been studies indicating that children’s unsociability was associated with poorer socio-emotional functioning in China. Although some researchers have found that parenting behavior would influence the relationship between children’s unsociability and adjustment, the role of parental psychological control has not been explored. This study aimed to investigate the moderating effect of parental psychological control on the relationship between unsociability and socio-emotional functioning in Chinese children.

**Methods:**

A total of 1,275 students from Grades 3 to 7 (637 boys, *M*_*age*_ = 10.78 years, *SD* = 1.55 years) were selected from four public schools in Shanghai to participate in this study. Data of unsociability, peer victimization and social preference were collected from peer-nominations, and data of parental psychological control, depressive symptoms and social anxiety were collected from self-reports.

**Results:**

There were positive associations between unsociability and peer victimization, depressive symptoms, and social anxiety, as well as a negative association between unsociability and social preference. Parental psychological control moderated these associations, specifically, the associations between unsociability and peer victimization, social preference, and depressive symptoms were stronger, and the association between unsociability and social anxiety was only significant among children with higher level of parental psychological control.

**Discussion:**

The findings in the current study highlight the importance of parental psychological control in the socio-emotional functioning of unsociable children in the Chinese context, enlightening educators that improving parenting behavior is essential for children’s development.

## Introduction

Social withdrawal refers to the process by which individuals refrain themselves from opportunities to interact with peers and frequently exhibit solitary behaviors in social contexts (Rubin and Coplan, 2004). According to the social approach-avoidance motivation model, there are two opposing motivational tendencies, social approach and social avoidance, and different combinations of them contribute to three subtypes of social withdrawal, including shyness, unsociability, and social avoidance (Asendorpf, 1990). Among these three subtypes of social withdrawal, unsociability is characterized by both low social approach and low social avoidance motivational tendencies, which differentiates it from shyness (i.e., high social approach and high social avoidance motivational tendencies) and social avoidance (i.e., low social approach and high social avoidance motivational tendencies). It has been found that the associations between different subtypes of social withdrawal and psychosocial adjustment were different (Coplan et al., 2015).

Unsociability is described as a non-fearful preference for solitary activities (Coplan and Armer, 2007). Previous studies have found that compared to in individualistic societies, children might experience higher adjustment difficulties in collectivistic societies (Rubin and Coplan, 2004; Liu et al., 2015), where group affiliation and social connectedness are highly valued (Chen, 2009). For example, there have been studies indicating that unsociability was associated with psychosocial maladjustment (e.g., depressive symptoms, loneliness and peer victimization) for children in China (Ding et al., 2015; Xiao et al., 2021; Zhao et al., 2023), where collectivism is the mainstream value (Chen, 2010). Given the possible risk of psychosocial adjustment among unsociable children in China, an important question arises as to whether certain factors may exacerbate or protect these children from developing maladjustment. Although there have been studies indicating that parents’ characteristics or behavior would influence the relationship between unsociability and children’s adjustment (Chen and Santo, 2016; Zhu et al., 2021), the role of parental psychological control, which is defined as parents’ control of their children’s inner state or behavior through love withdrawal, guilt induction or shaming (Yu et al., 2015), has not been explored. Therefore, the present study aimed to explore if parental psychological control would play a role in the relationship between children’s unsociability and socio-emotional functioning.

### Unsociability and socio-emotional functioning in Chinese children

According to the social learning perspective proposed by Hartup (1983), children acquire social skills and accomplish developmental tasks through interacting with peers. Given that unsociable children have low social approach motivational tendencies, they may experience inadequate peer interaction (Youniss, 1980; Rubin and Mills, 1988), which in turn increases their risk of socio-emotional difficulties (Rubin et al., 2009). However, given unsociability may be regarded as a personal choice and freedom in western societies, where individualism is the core value (Chen, 2019), it may have less negative influence on children’s adjustment in western societies. It has been found that unsociability was positively associated with attention span, and negatively associated with indexes of emotion dysregulation in western countries (Coplan et al., 2004). In contrast, in collectivist cultures, unsociability is viewed as deviating from the social values of group affiliation and belonging (Liu et al., 2017; Zhang and Eggum-Wilkens, 2018). Indeed, there have been studies indicating that unsociability was associated with negative developmental outcomes in Chinese children (Ding et al., 2015; Bullock et al., 2020; Xiao et al., 2021). A cross-national study has demonstrated that the associations between unsociability and children’s maladjustment were stronger in China than in Canada (Liu et al., 2015).

### The relationship between psychological control and children’s socio-emotional functioning

Given that group affiliation is a big concern when it comes to measuring social adjustment in China, Chinese parents may experience more stress when dealing with unsociable children in China. It has been found that parents tended to adopt less authoritative and more authoritarian parenting behavior toward their own child (Zhu et al., 2021). Psychological control is a parenting style in which parents use forms of psychological pressure and manipulation to control their children’s thoughts, feelings, and behaviors (Barber and Harmon, 2002). According to Self-determination Theory (Deci and Ryan, 2000), individuals have three kinds of basic psychological needs, including needs for autonomy, competence, and relatedness. Moreover, satisfaction of basic psychological needs can strengthen intrinsic motivation and internalization of extrinsic motivation in activities, contributing to people’s adjustment and development. However, parental psychological control is conflicted with children’s basic psychological needs. Previous studies have found that parents’ practice of psychological control is linked to children’s development of internalizing problems and social maladjustment (Barber and Harmon, 2002; Yu et al., 2015). Research from Western cultures has consistently shown that psychological control was associated with negative developmental outcomes, including depressive symptoms and anxiety among children and adolescents (Barber et al., 2005).

In contrast to the implied manipulative and authoritarian connotation of psychological control in Western societies, in China, parents usually consider psychological control as a necessary method to guide children to behave in ways that are consistent with the society’s value (Fung, 1999). This may be because in traditional Chinese culture, it is believed that children should show obedience and respect to their own parents (Liu et al., 2018), therefore parents’ behavior of control is regarded as a type of “training” for their children’s development (Chao, 1994). Despite this, growing evidence has indicated that psychological control was also linked to adjustment problems among children in East Asian contexts (Yu et al., 2015; Sun et al., 2017). There has been a recent study of need-supportive parenting program, showing that children would be more willing to participating in physical activities when their parents gave more support for their psychological need rather than controlling them (Meerits et al., 2023). As such, it is essential to explore the negative effect of parental psychological control, and reduce it in the real life.

### Theory foundation

According to the Goodness of Fit Theory (Thomas and Chess, 1977), children would have optimal developmental outcomes when their temperament is a good match for their parents’ parenting style. Given that unsociable children are characterized as preferring for solitary activities (Coplan and Armer, 2007), they may enjoy individual freedom and be reluctant to be restricted by others. As such, it can be speculated that for unsociable children, who have a higher need for autonomy, high parental psychological control is more detrimental for their adjustment than other children. It has been found that children’s unsociability would contribute to more adjustment problems, including peer exclusion, asocial behavior and anxious-fearful behavior when their parents adopted more authoritarian parenting behavior (Zhu et al., 2021). Moreover, it has been found that shy children would suffer from more internalizing problems or peer difficulties when they perceived higher parental psychological control (Bullock et al., 2018). However, as different subtypes of social withdrawal, children of unsociability have different social motivational tendencies compared to their peers of shyness. Therefore, it is essential to explore the role of parental psychological control in their adjustment and development.

### The present study

The primary aim of the present study was to explore the moderating effect of parental psychological control on the relationship between unsociability and socio-emotional functioning among Chinese children. Based on existing theoretical and empirical literature, we hypothesized that unsociability would be associated with socio-emotional problems. Furthermore, we speculated that parental psychological control would moderate the links between children’s unsociability and socio-emotional difficulties. Specifically, we anticipated that unsociability would have stronger associations with indices of socio-emotional difficulties among children who perceived higher level of parental psychological control. Given that previous research has found that the negative influence of unsociability on socio-emotional functioning might be more pronounced in boys than in girls (Liu et al., 2014), and in early adolescence than in childhood (Coplan et al., 2019), gender and grade were included in the models as control variables.

## Materials and methods

### Participants

Participants in current study were recruited from two primary schools and two secondary schools in Shanghai, People’s Republic of China. The total sample is comprised of 1,275 students (637 boys, 50% of all students) with a mean age of 10.78 years (*SD* = 1.55 years). The students were from Grades 3 to 7, and the information of students from different grades was displayed in Table 1. Almost all participating students were of Han nationality, which is the predominant nationality (over 90% of the population) in China (National Bureau of Statistics of China, 2018). Moreover, 94% of participants were from intact families, and approximately 49% of fathers and 40% of mothers had obtained a college education or higher.

**TABLE 1:** Information of students from different grades.

Grade	Number of students	Number of boys (percentage)	*M*_age_ (year)	*SD*_age_ (year)
3	280	132 (47.14%)	8.47	0.53
4	293	146 (49.83%)	9.43	0.54
5	267	135 (50.56%)	10.38	0.64
6	198	104 (52.53%)	11.56	0.72
7	237	120 (50.63%)	12.47	0.64

### Procedure

We conducted our original research by following steps. The design of the present study was reviewed and approved by the institutional review board of East China Normal University, and written informed consent was obtained from both participating students and their parents prior to data collection. During school hours, participating children completed self-report measures assessing their levels of depressive symptoms, social anxiety, and perceived paternal and maternal psychological control. These children also completed peer-nomination assessments, which included measures on unsociability, victimization, and social preference. The data collection process was carried out by a team of well-trained graduate research assistants, and each child was given a class list for the purpose of finishing the peer-nomination assessments more smoothly. The duration of the data collection process was approximately 30 min. To ensure the confidentiality of the participants’ responses, we assured them that their answers would be kept confidential. As a token of appreciation, each participating student received a small gift after submitting their completed questionnaire. The current study was conducted in 2013.

### Measures

#### Unsociability

Children’s unsociability was measured by the *Revised Class Play* (RCP; Masten et al., 1985; Chen, 1992) via peer nominations. Four items (e.g., “Someone who prefers playing alone,” “Someone who is reluctant to talking with others”) were included in this part. Each participant could nominate up to three classmates on each item, and as suggested by Terry and Coie (1991), both same-sex and cross-sex nominations were allowed. For each child, the nominations received on each item were standardized within classroom and summed, and standardized within classroom again to obtain the score of unsociability. This *RCP* measure have proved to be reliable and valid among Chinese children (Liu et al., 2014). In the present study, the internal consistency of this measure was α = 0.86.

#### Peer victimization

Peer victimization was assessed via peer nominations (Schwartz et al., 2001). Children nominated up to three peers to fit each of the seven items, which described physical victimization (e.g., “Someone who is always beat by others”), verbal victimization (e.g., “Someone who is always called names by others”) and relational victimization (e.g., “Someone others do not talk to”). For each child, the nominations received on each item were standardized within classroom and summed, and standardized within classroom again to obtain the score of peer victimization. The reliability and validity of this measure have been established in Chinese children (Liu et al., 2018). The internal consistency of this measure was α = 0.86 in this study.

#### Social preference

Following the procedure of Coie et al. (1982), each child was asked to nominate up to three classmates whom they most liked, and whom they least like in their classroom, respectively. The index of social preference was calculated by subtracting the standardized score of “like least” from the standardized score of “like most,” and standardizing the total score within classroom again. This measure has been demonstrated suitable for Chinese children (Chen et al., 1995).

#### Depressive symptoms

The level of depressive symptoms was measured using the Chinese version of *Children’s Depression Inventory* (CDI; Kovacs, 1992) via self-report. The CDI is comprised of 14 items that assess children’s depressive mood over the past 2 weeks. Participants were instructed to choose one sentence which best described their own internal state (e.g., “I like myself,” “I do not like myself,” “I hate myself”). The items were rated on a 3-point scale, with a higher average score indicating a higher level of depressive symptoms. This measure has been shown to be reliable and valid in Chinese children (Liu et al., 2019). In the current study, the internal consistency of this measure was α = 0.83.

#### Social anxiety

Children’s level of social anxiety was assessed using the Chinese version of Social Anxiety Scale for Children-Revised (SASC-R; La Greca and Stone, 1993) via self-report. The scale consisted of 15 items that measured fear of negative evaluation (e.g., “I worry about what other kids think of me”), social avoidance and distress to new peers or situations (e.g., “I get nervous when I talk to new kids”), and general social avoidance and distress (e.g., “I am quiet when I am with a group of kids”), respectively. Participants rated their response on a five-point Likert scale, with higher average scores indicating greater level of social anxiety. The reliability and validity of this measure have been demonstrated in Chinese children (Gao et al., 2022). In the current study, the internal consistency of this measure was α = 0.90.

#### Parental psychological control

Children reported on how often their mothers and fathers engage in psychologically controlling parenting on a five-point Likert scale (Yu et al., 2015). The scale consisted of 18 items that measure love withdrawal (e.g., “My father/mother would avoid looking at me when I disappointed him/her”), guilt induction (e.g., “My father/mother would make me aware of how much he/she sacrificed or did for me”) and shaming of children (e.g., “My father/mother would tell me that I should be ashamed when I misbehaves”). Given that the paternal and maternal psychological control were highly correlated (*r* = 0.81, *p* < 0.001), we averaged mother’s and father’s data of each child to form the indicator of parental psychological control. Previous studies have proved that this measure was reliable and valid for Chinese children (Bullock et al., 2018). In the current study, the internal consistency of this measure was α = 0.91.

### Data analysis

We handled the missing data by the full information maximum likelihood method (Graham, 2009) with the MLR estimation in *Mplus* 7.4. The steps of analyzing data were as follow. To begin with, we conducted descriptive statistics using IBM SPSS for Windows (version 25). The Pearson correlation coefficients among the study variables were calculated. Additionally, we conducted a two-way multivariate analysis of variance (MANOVA) and four two-way analyses of variance (ANOVA) to examine the effects of gender (boys = 0, girls = 1) and grade (primary school = 0, secondary school = 1) on the study variables. Next, we used *Mplus* version 7.4 (Muthén and Muthén, 2012) to conduct moderation analyses for peer victimization, social preference, depressive symptoms and social anxiety, respectively. Finally, the simple slope test would be conducted (Aiken and West, 1991) when significant moderating effect was found. Parental psychological control, gender and grade were grand-mean centered before the data analysis.

## Results

### Missing data

For the self-reported variables, which included depressive symptoms, social anxiety and parental psychological control, the percentage of missing data was 3.5, 3.5, and 3.2%, respectively. The Little’s MCAR test (Little, 1988) was significant [χ^2^(9977) = 12116.21, *p* < 0.001]. However, following Ulman (2013) recommendation, χ^2^/df = 1.21 < 2, suggesting that the missing data can be considered missing completely at random.

### Descriptive statistics

The intercorrelations, means and standard deviations of study variables are shown in Table 2. Unsociability was positively associated with peer victimization, depressive symptoms and social anxiety, and negatively associated with social preference. Parental psychological control was positively associated with peer victimization, depressive symptoms and social anxiety, and negatively associated with social preference.

**TABLE 2:** Descriptive statistics of study variables.

	1	2	3	4	5	6
1 Unsociability						
2 Peer victimization	0.54***					
3 Social preference	−0.42***	−0.65***				
4 Depressive symptoms	0.28***	0.28***	−0.27***			
5 Social anxiety	0.07*	0.06	−0.09**	0.31***		
6 Parental psychological control	0.05	0.13***	−0.15***	0.23***	0.23***	
*M*	0.00	0.00	0.00	1.37	2.22	2.46
*SD*	0.99	0.99	0.98	0.32	0.84	0.64

*p < 0.05;

**p < 0.01;

***p < 0.001.

The multivariate analysis of variance (MANOVA) revealed significant main effect of gender [Wilk’s λ = 0.93, *F*_(6, 1187)_ = 15.00, *p* < 0.001, partial η^2^ = 0.07] and grade [Wilk’s λ = 0.96, *F*_(6, 1187)_ = 8.17, *p* < 0.001, partial η^2^ = 0.04]. However, the interaction between gender and grade was not significant. Further analysis of variance (ANOVA) showed that girls scored higher on social preference[*F*_(1, 1261)_ = 27.18, *p* < 0.001, partial η^2^ = 0.02] and social anxiety [*F*_(1, 1226)_ = 10.18, *p* = 0.001, partial η^2^ = 0.01], but scored lower on unsociability [*F*_(1, 1261)_ = 6.30, *p* = 0.012, partial η^2^ = 0.01], peer victimization [*F*_(1, 1261)_ = 45.01, *p* < 0.001, partial η^2^ = 0.03] and parental psychological control [*F*_(1, 1230)_ = 33.61, *p* < 0.001, partial η^2^ = 0.03] than boys, and students from primary school scored lower on depressive symptoms [*F*_(1, 1227)_ = 38.13, *p* < 0.001, partial η^2^ = 0.03] and parental psychological control [*F*_(1, 1230)_ = 13.57, *p* < 0.001, partial η^2^ = 0.01] compared to students from secondary school. The means and standard deviations of study variables of different gender and grade are shown in Table 3.

**TABLE 3:** Means and standard deviations of study variables of different gender and grade (*M* ± *SD*).

	Primary schools		Secondary schools	
	**Boys**	**Girls**	**Boys**	**Girls**
Unsociability	0.11 ± 1.02	−0.10 ± 0.95	0.04 ± 0.95	−0.04 ± 1.02
Peer victimization	0.23 ± 1.15	−0.22 ± 0.75	0.16 ± 1.05	−0.17 ± 0.88
Social preference	−0.15 ± 1.06	0.15 ± 0.88	−0.15 ± 1.02	0.16 ± 0.92
Depressive symptoms	1.37 ± 0.30	1.30 ± 0.29	1.45 ± 0.34	1.45 ± 0.37
Social anxiety	2.15 ± 0.79	2.26 ± 0.87	2.15 ± 0.86	2.36 ± 0.83
Parental psychological control	2.52 ± 0.64	2.30 ± 0.62	2.66 ± 0.64	2.44 ± 0.63

### Moderation analyses

Moderating effects of parental psychological control on the relationships between unsociability and each indicator of socio-emotional adjustment were examined. The results are shown in Table 4.

**TABLE 4:** The moderation analyses of parental psychological control on psychosocial adjustment.

	Peer victimization		Social preference		Depressive symptoms		Social anxiety	
	***b***	***SE***	***b***	***SE***	***B***	***SE***	***b***	***SE***
Unsociability	0.53***	0.02	−0.41***	0.03	0.09***	0.01	0.05*	0.02
Gender	−0.28***	0.05	0.18***	0.05	−0.01	0.02	0.23***	0.05
Grade	−0.02	0.05	0.04	0.05	0.11***	0.02	0.01	0.05
Control	0.13***	0.04	−0.18***	0.04	0.10***	0.01	0.33***	0.04
Unsociability × Gender	−0.05	0.05	−0.10	0.05	−0.01	0.02	0.10	0.05
Unsociability × Grade	0.11*	0.05	−0.04	0.05	0.05*	0.02	−0.01	0.05
Unsociability × Control	0.09**	0.03	−0.08*	0.04	0.03*	0.01	0.08*	0.03

Control, parental psychological control.

*p < 0.05;

**p < 0.01;

***p < 0.001.

**FIGURE 1:**
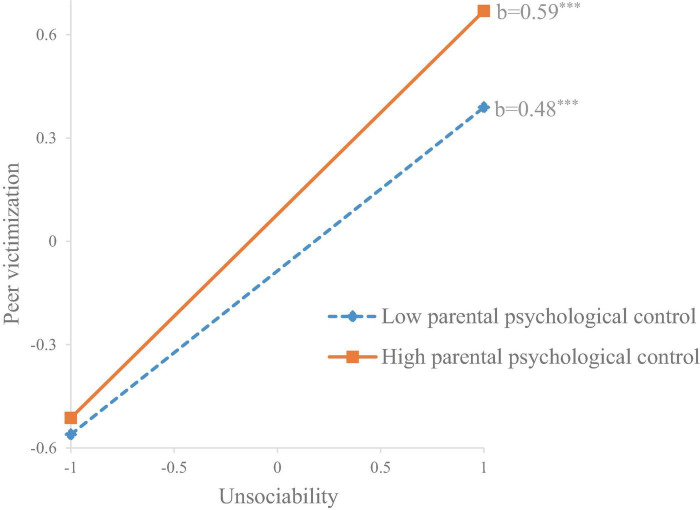
The moderating effect of parental psychological control on the association between unsociability and peer victimization. ***p < 0.001.

In terms of peer victimization, the finding indicated that parental psychological control had a significant moderating effect on the positive association between unsociability and peer victimization (*b* = 0.09, *SE* = 0.03, *p* = 0.008). Specifically, this association was stronger when children perceived higher parental psychological (M + 1 SD) (*b* = 0.59, *SE* = 0.03, *p* < 0.001) than when they perceived lower parental psychological control (M–1 SD) (*b* = 0.48, *SE* = 0.03, *p* < 0.001) (see Figure 1). Additionally, grade was found to significantly moderate the association between unsociability and peer victimization (*b* = 0.11, *SE* = 0.05, *p* = 0.021). Specifically, this association was stronger in students from secondary schools (*b* = 0.61, *SE* = 0.04, *p* < 0.001) compared to students from primary schools (*b* = 0.49, *SE* = 0.03, *p* < 0.001). The moderating effect of gender was insignificant.

**FIGURE 2:**
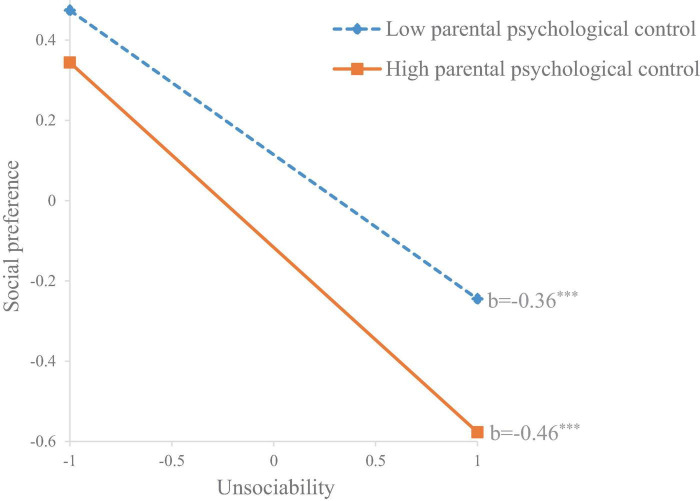
The moderating effect of parental psychological control on the association between unsociability and social preference. ***p < 0.001.

For social preference, parental psychological control also moderated the significant negative association between unsociability and social preference (*b* = −0.08, *SE* = 0.04, *p* = 0.033). Specifically, this association was stronger in children who perceived higher parental psychological (*b* = −0.46, *SE* = 0.03, *p* < 0.001) compared to those who perceived lower parental psychological control (*b* = −0.36, *SE* = 0.04, *p* < 0.001) (see Figure 2). The moderating effects of gender and grade were insignificant.

**FIGURE 3:**
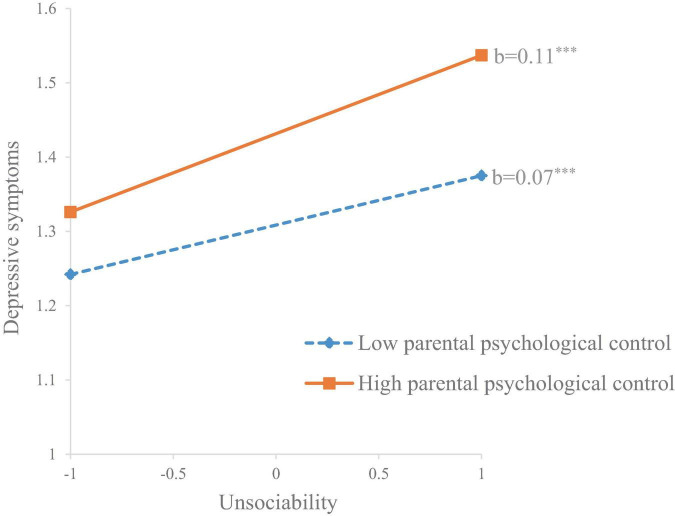
The moderating effect of parental psychological control on the association between unsociability and depressive symptoms. ***p < 0.001.

For depressive symptoms, parental psychological control also moderated the positive association between unsociability and depressive symptoms (*b* = 0.03, *SE* = 0.01, *p* = 0.013). Specifically, this association was stronger in children who perceived higher parental psychological (*b* = 0.11, *SE* = 0.01, *p* < 0.001) compared to those who perceived lower parental psychological control (*b* = 0.07, *SE* = 0.01, *p* < 0.001) (see Figure 3). Additionally, grade was found to significantly moderate the association between unsociability and depressive symptoms (*b* = 0.05, *SE* = 0.02, *p* = 0.012). Specifically, this association was stronger in students from secondary schools (*b* = −0.12, *SE* = 0.02, *p* < 0.001) compared to students from primary schools (*b* = 0.07, *SE* = 0.01, *p* < 0.001). The moderating effect of gender was insignificant.

**FIGURE 4:**
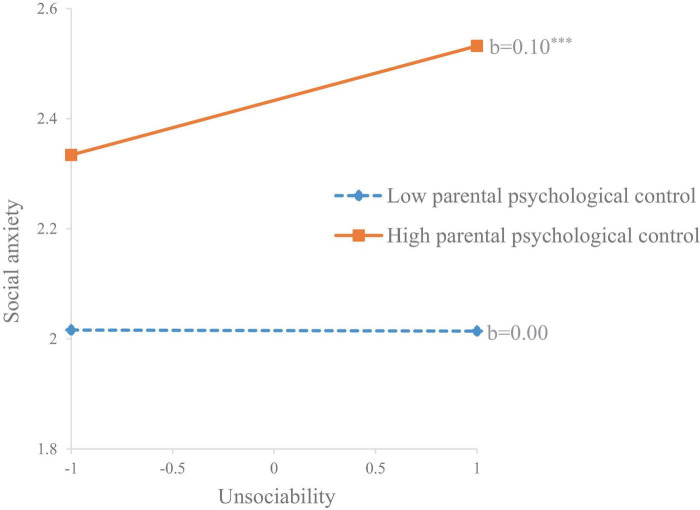
The moderating effect of parental psychological control on the association between unsociability and social anxiety. ***p < 0.001.

For social anxiety, parental psychological control also moderated the positive association between unsociability and social anxiety (*b* = 0.08, *SE* = 0.03, *p* = 0.023). Specifically, this association was only significant when children perceived higher parental psychological (*b* = 0.10, *SE* = 0.03, *p* = 0.001) rather than when they perceived lower parental psychological control (*b* = 0.00, *SE* = 0.04, *p* = 0.984) (see Figure 4). The moderating effects of gender and grade were insignificant.

## Discussion

This study aimed to investigate the association between unsociability and socio-emotional functioning in Chinese children, and to explore the potential moderating effect of parental psychological control on this relationship. Our findings first supported the hypothesis that unsociability is associated with adjustment problems in urban China today. Furthermore, parental psychological control was found to moderate the relationship between unsociability and socio-emotional functioning. Specifically, our results showed that unsociable behaviors of children are linked more profoundly to peer victimization and low preference by peers when children perceived higher levels of psychological control from their parents. Similarly, our results also indicated that unsociability is associated more closely with internalizing outcomes (i.e., depressive symptoms and social anxiety) in children with higher levels of parental psychological control compared to children whose parents are less controlling. These results have important implications for understanding the role of parental psychological control in the relationship between unsociability and socio-emotional functioning in Chinese children.

### The moderating effects of parental psychological control on unsociable children’s socio-emotional functioning

It is well established that unsociability is associated with less prosocial behaviors and lower social interactive skills (Rubin et al., 2009), contributing to children’s poor developmental outcomes. In China, a country where children who are not interested in group interactions would be regarded as selfish and deviant (Liu et al., 2017), unsociable children were found to be more susceptible to internalizing problems (Bullock et al., 2020) as well as peer difficulties (Liu et al., 2014). The results in our study further highlight this phenomenon.

Our findings are consistent with previous research that highlighted the importance of favorable parenting style in improving mental health and reducing the risk of socio-emotional difficulties among socially withdrawn children and youths (Degnan and Fox, 2007; Zarra-Nezhad et al., 2014). Parental psychological control is a dimension of parenting style that can impact children’s socio-emotional development. A high level of psychological control, characterized by behaviors such as manipulation, guilt induction, and love withdrawal (Barber, 1996), may increase the risk of youths’ problem behaviors (Yan et al., 2020). The perspective of social support may explain for why parental psychological control would strengthen the associations between children’s unsociability and maladjustment. Seeking social support is one type of coping strategies while confronting with difficulties (Causey and Dubow, 1992), and there have been studies indicating that social support from others (e.g., parents, friends and teachers) was beneficial for children’s adjustment in school (Rueger et al., 2010; Fan and Fan, 2021). It has been found that friend’s support could weaken the associations between unsociability and internalizing problems (Barstead et al., 2018). However, for unsociable children who are characterized as preferring spending time alone (Coplan and Armer, 2007), they may lack social support from their friends, and support from their parents may compensate for it. Therefore, compared to other children, unsociable children may have a stronger need of their parents’ support. Moreover, according to Goodness of Fit Theory (Thomas and Chess, 1977), unsociable children may be more sensitive to controlling parenting behavior because of their own temperament. Influenced by parental psychological control, such an unsupportive parenting style, unsociable children would feel depressed and anxious in their daily lives. Furthermore, they can’t learn social skills from their parents of high controlling parenting behavior, thus it is hard for them to build good relationships with others, which may explain why they had higher level of peer victimization and lower level of social preference when they perceived high parental psychological control.

Our study also shows that age moderates the associations between unsociability and peer victimization and depressive symptoms, with a stronger relationship observed in secondary schools than primary schools. The possible reason might be many other factors outside of family settings, such as peer or teacher, have a growing impact on children as they enter adolescence (Lamblin et al., 2017). Indeed, the social expectations of having more peer interactions increase at peak in early adolescence (Coplan et al., 2019). Therefore, compared to primary school students, secondary school students’ unsociable behavior may be regarded as more unacceptable, because they violate the recognized social norm. As a result, secondary school students are more likely to be excluded by their peers and suffer from socio-emotional difficulties if they have high level of unsociability.

### Limitations and future directions

The present study provided insights on the role of parental psychological control in the links between unsociability and socio-emotional functioning among Chinese children. Nevertheless, there are several limitations to this study. First, our study was cross-sectional in nature and it was hard to infer the results of causalities. Future research can utilize longitudinal research with multiple waves to further infer causal effects. Second, children’s unsociability was assessed through peer nominations (Chen, 1992). Given that unsociability was characterized as a combination of low social-approach motivational tendency and low social-avoidance tendency (Asendorpf, 1990), peer nominations of it may not reflect children’s own motivation. Future research can measure children’s unsociability through self-report. Third, parental psychological control was measured via children’s self-reports (Yu et al., 2015), which might not fully reflect actual parenting behavior. Future research should evaluate parental psychological control through observational methods and gather data from parents’ reports. Fourth, some coefficients in the results are significant but small, probably because the sample size of the current study is large. Therefore, the explanation of the results should be cautious. Finally, our study was conducted in Shanghai, a large city in China. Due to the huge differences in the rural and urban areas in China, the results might not be applicable to rural areas. Moreover, the applicability of the results should be also examined in western countries, where parental psychological control may exert different influence.

Despite these limitations, our study reveals the moderating role of parental psychological control between unsociability and socio-emotional functioning among children. For theoretical implications, the results indicate that parental psychological control, a common parenting style in Eastern Asian cultures, might impact children’s adjustment and development in a more subtle and nuanced way. Moreover, the results also suggest that unsociable children and youth could be more susceptible to controlling parenting. For the practical implications, the current study can inform school educators when they hold parenting workshops. In these parenting workshops, educators are encouraged to inform parents that it is important to focus on their children’s psychological needs, such as need for autonomy and need for relatedness, and provide more support for them rather than exhibiting need-thwarting behavior (Ahmadi et al., 2023), especially in China, where controlling parenting behavior is regarded as relative normal and effective. Moreover, society and school can highlight the importance of supporting parents of unsociable children, as their behaviors seemed to have a more profound impact on their children’s adaptation.

## Conclusion

This study was aimed at exploring the moderating effect of parental psychological control on the relationship between unsociability and socio-emotional functioning in Chinese children. In the sample of the study, unsociability had a significant positive association with peer victimization, depressive symptoms, and social anxiety, as well as a significant negative association with social preference. The moderating effect of parental psychological control was significant on all of four associations. Specifically, the associations between unsociability and peer victimization, social preference, and depressive symptoms were stronger, and the association between unsociability and social anxiety was only significant among children with higher level of parental psychological control. The results indicated that parental psychological control was a risk factor for unsociable children’s adjustment in China.

## Data availability statement

The raw data supporting the conclusions of this article will be made available by the authors, without undue reservation.

## Ethics statement

The studies involving humans were approved by the Research Ethics Review Board at East China Normal University. The studies were conducted in accordance with the local legislation and institutional requirements. Written informed consent for participation in this study was provided by the participants’ legal guardians/next of kin.

## Author contributions

HZ: Conceptualization, Funding acquisition, Supervision, Writing – original draft. YH: Formal Analysis, Methodology, Writing – original draft, Writing – review and editing. YC: Data curation, Formal Analysis, Methodology, Writing – review and editing. RL: Investigation, Validation, Writing – review and editing. NW: Data curation, Validation, Writing – review and editing. XC: Methodology, Writing – review and editing. TC: Conceptualization, Supervision, Writing – review and editing. JL: Conceptualization, Funding acquisition, Investigation, Supervision, Writing – review and editing.
